# Filoviral Immune Evasion Mechanisms

**DOI:** 10.3390/v3091634

**Published:** 2011-09-07

**Authors:** Parameshwaran Ramanan, Reed S. Shabman, Craig S. Brown, Gaya K. Amarasinghe, Christopher F. Basler, Daisy W. Leung

**Affiliations:** 1 Department of Biochemistry, Biophysics and Molecular Biology, Iowa State University, Ames, IA 50011, USA; 2 Department of Microbiology, Mount Sinai School of Medicine, New York, NY 10029, USA; 3 Biochemistry Graduate Program, Iowa State University, Ames, IA 50011, USA; 4 Biochemistry Undergraduate Program, Iowa State University, Ames, IA 50011, USA

**Keywords:** *Ebolavirus*, *Marburgvirus*, IFN antagonist, innate immune evasion

## Abstract

The *Filoviridae* family of viruses, which includes the genera *Ebolavirus* (EBOV) and *Marburgvirus* (MARV), causes severe and often times lethal hemorrhagic fever in humans. Filoviral infections are associated with ineffective innate antiviral responses as a result of virally encoded immune antagonists, which render the host incapable of mounting effective innate or adaptive immune responses. The Type I interferon (IFN) response is critical for establishing an antiviral state in the host cell and subsequent activation of the adaptive immune responses. Several filoviral encoded components target Type I IFN responses, and this innate immune suppression is important for viral replication and pathogenesis. For example, EBOV VP35 inhibits the phosphorylation of IRF-3/7 by the TBK-1/IKKε kinases in addition to sequestering viral RNA from detection by RIG-I like receptors. MARV VP40 inhibits STAT1/2 phosphorylation by inhibiting the JAK family kinases. EBOV VP24 inhibits nuclear translocation of activated STAT1 by karyopherin-α. The examples also represent distinct mechanisms utilized by filoviral proteins in order to counter immune responses, which results in limited IFN-α/β production and downstream signaling.

## 1. Filoviruses

Filoviruses are single-stranded, negative sense RNA viruses, which cause severe viral hemorrhagic fever in humans and non-human primates. *Ebolaviruses* (EBOV) and *Marburgviruses* (MARV) comprise the *Filoviridae* family. Five species of EBOV (Zaire, Sudan, Cote-d’Ivoire, Reston, Bundibugyo) and one species of MARV (Lake Victoria) have currently been identified. Among the five species of EBOV, Reston *Ebolavirus* (REBOV) does not cause disease in humans, although it is highly fatal to non-human primates [1,2]. Several patients tested seropositive for Cote-d’Ivoire species (CIEBOV), and displayed disease symptoms characteristic of hemorrhagic fever. However, no fatalities were directly attributed to CIEBOV. In contrast, REBOV was initially identified in an outbreak at an animal handling facility in Reston, Virginia, among macaques imported from the Philippines [2]. All of the macaques suffered from viral hemorrhagic fever resulting in death, while the two animal handlers who came in to contact with the animals had seroconverted. However, they did not display any disease symptoms. More recently, REBOV has been isolated from a swine population in the Philippines, whose animal handlers were seropositive, suggesting that the zoonotic nature of filoviruses may potentially make filoviruses a serious public health threat [2,3]. These isolated, but significant incidents highlight the potential threat of filoviruses worldwide. Currently limited information is known about the range of hosts that filoviruses infect and the virulence factors that promote zoonosis and lateral disease transfer (animal to animal). Studies to date, including some recent field studies indicate that several different species of fruit bats may serve as the natural reservoir for filoviruses [4–6]. The lethality of filoviral infections have been attributed to the ability of the virus to efficiently suppress the host innate antiviral responses early during infection, followed by impairment and/or dysregulation of the adaptive immune response and inflammatory pathways late during infection [7]. This is proposed to be due in large part to viral replication within dendritic cells, monocytes, macrophages, and hepatocytes [8]. Currently, the mechanisms involving host immune modulation by filoviral components are not clearly understood. In this review, we primarily focus on the following filoviral proteins: EBOV viral protein (VP) 35, EBOV VP24, and MARV VP40. Below we discuss current biochemical, structural and cell biological data and the mechanisms by which these filoviral proteins modulate the host innate immune responses. Having such information at hand will provide a starting point for developing countermeasures against filoviral infections.

## 2. Host Innate Immune Mechanisms

**Figure 1.**
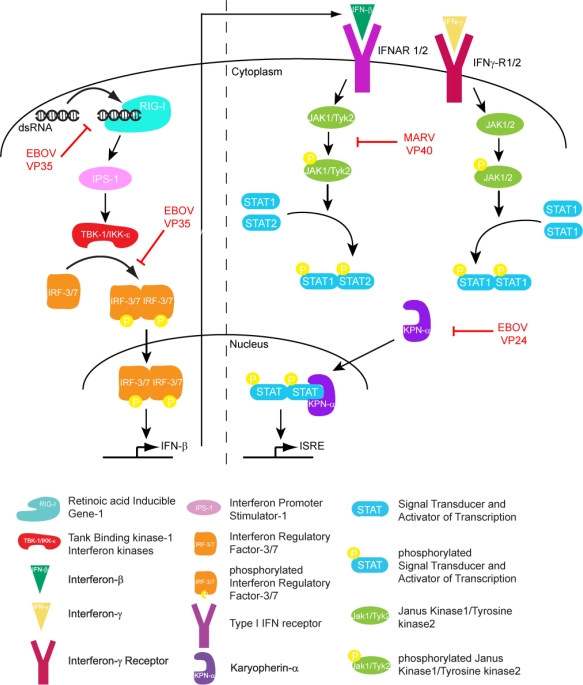
Filoviral proteins counter the host IFN response through multiple mechanisms in order to limit host antiviral responses.

The host innate immune response is triggered upon recognition of pathogen associated molecular patterns (PAMPs) by pattern recognition receptors (PRRs), which are located both on the surface and in the cytoplasm of cells. Although both Toll-like receptors (TLRs) and RIG-I like receptors (RLRs) play key roles in activating innate immune response, here we will primarily focus on RLRs as recent literature suggest that filoviruses antagonize innate immune responses downstream of RLRs. RLRs, which include retinoic acid inducible gene-I (RIG-I) and melanoma differentiation associated gene-5 (MDA5), are cytoplasmic sensors of viral infections. These receptors are likely regulated by autoinhibition, which prevents effector access to the N-terminal caspase activation and recruitment domains (CARDs) and helicase activity [9,10]. Upon activation, RLRs signal through the adaptor molecule IPS-1, which activates the kinases Tank binding kinase-1 (TBK-1) and I-Kappa-B kinase epsilon (IKKε). TBK-1/IKKε phosphorylate interferon regulatory factors (IRF) 3 and 7 [10]. Upon phosphorylation, IRF3/7 dimerize and localize to the nucleus, where they activate the transcription and production of interferon beta (IFN)-β (Figure 1). IFN-β can signal in both an autocrine and paracrine manner through interaction with its cognate receptor IFN alpha/beta receptor 1/2 (IFNAR1/2). Activation of IFNAR1/2 subsequently triggers the JAK/STAT signaling cascade, which involves the activation and autophosphorylation of Janus Kinase (JAK) family members JAK1 and tyrosine kinase 2 (TYK2), and the phosphorylation of signal transducer and activator of transcription (STAT) 1 and 2 proteins. Upon phosphorylation, STAT1/2 dimerize and localize to the nucleus where they activate the transcription of the interferon stimulated genes (ISGs), such as double-stranded RNA-dependent protein kinase (PKR), 2′-5′ oligoadenylate synthetase (OAS), RNAseL, RNA-specific adenosine deaminsases (ADARs), and MHC class 1 and 2 [11]. The successful activation and transduction of these signaling events lead to upregulation of these and other host antiviral genes, thus establishing an antiviral state in the cell. Not surprisingly, these key innate immune signaling molecules are targeted by many virally encoded proteins, including those encoded by filoviruses. Both EBOV and MARV infections have been shown to modulate the expression of genes that are involved in the host immune response. Transcriptional profiling of human liver cells infected with ZEBOV or MARV show that there are major changes in the host gene expression profiles within 24 to 48 hours post infection [12,13]. Many of these genes are involved in immune regulation, coagulation, and apoptosis pathways. Furthermore, these gene expression changes are likely associated with severe suppression of ISG production, indicating that both ZEBOV and MARV are able to efficiently abrogate IFN responses and display rapid replication kinetics [12,13]. In contrast, REBOV infected cells displayed slower viral replication kinetics and also displayed significantly higher ISG production [13]. Together, these data suggests that REBOV is comparatively less efficient in abrogating host IFN responses in humans. These potential differences in host gene expression patterns may also contribute to observed differences in host tropism between REBOV and ZEBOV. However, many of these aspects have not been characterized at the molecular level.

## 3. Filoviral Encoded Immune Antagonists

The filoviral genome is approximately 19 kilobases and encodes for at least seven structural proteins, including nucleoprotein (NP), viral protein (VP) 35, VP40, glycoprotein (GP), VP30, VP24, and the viral RNA-dependent RNA polymerase (L). With the recent identification of small soluble glycoprotein (sGP), there are at least three known variants of glycoprotein thus bringing the total number of proteins that can be potentially encoded by the EBOV genome to nine [14]. Each of these proteins is multifunctional and performs multiple roles during distinct stages in the viral lifecycle. For example, NP forms part of the nucleocapsid and is also a part of the replication complex along with VP30, VP35, and L. Of these filoviral proteins, EBOV VP35, EBOV VP24, and MARV VP40 have been recently implicated in host innate immune antagonism through a variety of mechanisms. Virally encoded GP and sGP have also been implicated in modulating immune responses, including a role for sGP as a decoy that can occupy host receptors and prevent recognition of viral membrane bound GP proteins. Below we focus on the various roles of EBOV VP35 and VP24 and MARV VP40 proteins and discuss how each contributes to filoviral evasion from host immune detection.

### 3.1. EBOV VP35 Inhibits IFN Production

EBOV VP35 was initially identified as a Type I IFN antagonist. Expression of VP35 functionally substituted for the influenza A virus NS1 protein, a known inhibitor of IFN α/β responses, in a growth complementation assay [15,16]. Specifically, VP35 rescued the growth defect of an influenza virus that lacked the NS1 protein in 293T cells, whereas expression of other EBOV proteins could not. Furthermore, VP35 suppressed IFN-β mRNA production and inhibited the activation of both IFN-β and ISG54 promoter induced by transfected polyI:C, mutant influenza virus infection, or by Sendai virus infection, suggesting that EBOV VP35 functions as an IFN antagonist similar to NS1.

**Figure 2.**
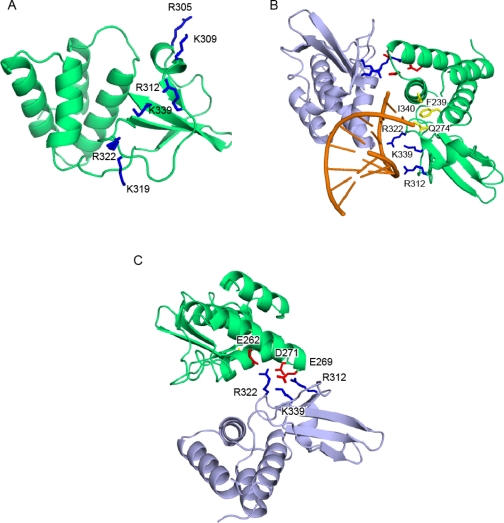
VP35 IID is involved in both protein-protein and protein-RNA interactions. (**A**) Residues within the CBP (dark blue) of VP35 IID (green) are important for dsRNA-dependent and -independent activities. (**B**) Residues that are involved in hydrophobic “end-cap” interactions with the dsRNA blunt ends are shown in yellow. CBP residues involved in recognition of the phosphate backbone of dsRNA (orange) are highlighted in dark blue. (**C**) Conserved CBP residues of one VP35 IID molecule (light blue) are also involved in protein-protein interactions with acidic residues of the neighboring molecule of VP35 IID (green) in the crystal structure.

**Figure 3.**
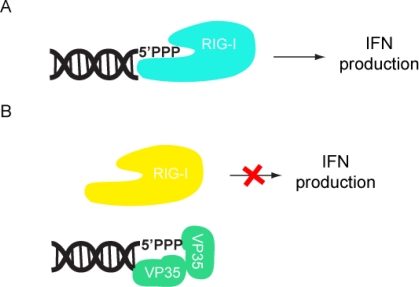
VP35 inhibits IFN production by sequestering viral RNA. (**A**) RIG-I is activated by RNA, including dsRNA, and triggers a signal transduction cascade leading to Type I IFN production. (**B**) VP35 IID mimics dsRNA recognition by the RNA-binding domain of RIG-I.

Subsequent studies showed that the C-terminus of VP35 makes a major contribution to the ability of VP35 to inhibit IFN production. Initial sequence alignments of ZEBOV VP35 with influenza NS1 revealed a short stretch of conserved amino acids, including basic residues Arg 305, Lys 309, and Arg 312. Mutation of these residues to alanine led to a loss of dsRNA binding and IFN inhibition [17,18]. In addition, a mouse-adapted EBOV containing a VP35 Arg 312 alanine mutant, was growth attenuated compared to the wildtype virus when transfected into macrophages or hepatocyte cells [19]. The crystal structures of the C-terminal IFN inhibitory domain (IID) of ZEBOV and REBOV VP35 were recently solved, both in the free and dsRNA-bound forms [20,21]. Comparison of ZEBOV VP35 IID with other RNA binding proteins show that VP35 IID forms a novel fold that recognizes and binds dsRNA in a sequence independent manner. VP35 IID contains two patches of basic residues. The first basic patch (FBP), including residues Lys 222, Arg 225, Lys 248, and Lys 251, is located on the α-helical subdomain of IID whereas the central basic patch (CBP), consisting of residues Arg 305, Lys 309, Arg 312, Lys 319, Arg 322 and Lys 339, is located on the β-sheet subdomain of IID (Figure 2A). Residues in the CBP, and not the FBP, are important for dsRNA binding, as these residues make direct contacts with the phosphate backbone of dsRNA (Figure 2B) [21]. In addition, the CBP residues Arg 312, Arg 322 and Arg 339 are also involved in protein-protein interactions with acidic residues Glu 262, Glu 269 and Asp 271 of the neighboring molecule of VP35 IID as observed in the crystal structure of VP35 IID-dsRNA complex (Figure 2C). These interactions could potentially play an important role in dsRNA-independent functions of VP35. Residues in the CBP of VP35 IID that are critical for dsRNA binding are also important for IFN inhibition. Mutation of CBP residues to alanine, especially Arg 312, suppresses VP35’s ability to inhibit IFN-β promoter activity [21]. The structure of ZEBOV VP35 IID bound to dsRNA also revealed that VP35 end caps the blunt ends of dsRNA (Figure 2B). Hydrophobic and base stacking interactions involving VP35 residues F239, I340 and Q274 with dsRNA likely compete with RIG-I like receptors (RLRs) for dsRNA binding. This provides a mechanism by which VP35 interferes and prevents the recognition of viral RNA by RLRs and activation of the IFN-β signaling pathway (Figure 3). Interestingly, recombinant EBOV containing VP35 with mutations within the CBP are avirulent in guinea pigs [22], while animals infected with recombinant virus containing the wildtype sequence succumb to the virus within 6–8 days. Moreover, exposure to a recombinant CBP mutant (incapable of binding dsRNA and inhibiting RIG-I activation) virus protected the animals from subsequent infection with wildtype recombinant virus [22].

Further analysis of the VP35 IID structure revealed that the CBP residues are also involved in protein-protein interactions. VP35 can partially suppress the dsRNA-independent activation of the IFN-β promoter as mutation of Arg 312, Arg 322, and Lys 339 results in the loss of IFN inhibition [21]. In addition to the CBP, the coiled-coil region of VP35 which facilitates VP35 oligomerization is also important for the IFN antagonist function of VP35 [23]. This suggests that the oligomerization motif on the amino-terminus facilitates the IFN antagonist activity on the carboxy-terminus.

**Figure 4.**
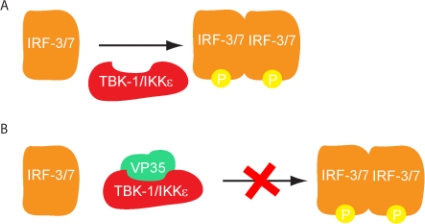
VP35 inhibits IFN kinase phosphorylation of IRF3/7. (**A**) Phosphorylation of IRF3/7 by TBK-1/IKKε leads to the dimerization and translocation of IRF3/7 into the nucleus where it activates the IFN-β promoter. (**B**) EBOV VP35 inhibits IRF-3/7 phosphorylation by interacting with TBK-1 and IKKε kinase domain.

VP35 also modulates the activity of several host components independent of its ability to bind dsRNA, in order to suppress IFN production. Earlier studies demonstrated that when EBOV VP35 transfected cells that are challenged with Sendai virus, IRF-3 localization to the nucleus is impaired [15]. However, VP35 cannot inhibit IFN-β promoter activity by a constitutively active IRF-3 mutant suggesting that VP35 inhibits components upstream of IRF-3 phosphorylation. VP35 also inhibits IFN-β promoter activation mediated by TBK-1/IKKε overexpression. These data strongly suggest that VP35 specifically inhibits the phosphorylation of IRF-3 by TBK-1/IKKε [15,24]. Furthermore, VP35 interacts with the kinase domain of TBK-1/IKKε which impairs the phosphorylation of IRF3/7 by TBK-1/IKKε [24]. Thus, VP35 likely serves as an alternate substrate for IFN kinases thus preventing IFN-β production (Figure 4).

In addition to suppression of RLR activation and RLR mediated IFN production, VP35 has been shown to antagonize several other antiviral mechanisms in the host cell. Recently, VP35 was shown to hijack the host SUMOylation machinery by interacting with SUMO E2 enzyme Ubc9, and the SUMO E3 ligase PIAS1 [25]. VP35 promoted the selective SUMOylation of IRF-7 thereby repressing IFN transcription. VP35 has also been shown to inhibit PKR mediated shut down of host translational machinery [26,27]. Herpes Simplex virus (HSV) encodes for a protein known as γ_1_34.5 (also called ICP34.5) which antagonizes PKR by recruiting host phosphatase to dephosphorylate eIF2α. Recombinant HSV which lacks this gene is unable to replicate in cells which can mount a robust immune response resulting in PKR upregulation and activation. However, transfecting VP35 into these cells rescued the recombinant virus replication and restored viral protein synthesis [26]. This was correlated to the suppression of PKR activity and reduced eIF2α phosphorylation. It is likely that the ability of VP35 to bind dsRNA may play role in its ability to inhibit PKR, although this hypothesis remains to be tested as some residues (*i.e.*, residues in the CBP) participate in both protein-protein and protein-RNA interactions. The dsRNA binding activity of VP35 has also been shown to be important for suppressing the host RNAi pathway. VP35 has been shown to functionally replace HIV-1 Tat protein, which is a well-established inhibitor of RNA silencing.[28]. VP35 was able to inhibit RNAi in a reporter gene assay at levels similar to influenza NS1 protein and vaccinia virus E3L protein, both of which are previously characterized viral RNAi suppressors. A more recent study has shown VP35 functions as an antagonist of RNAi [28,29]. Immunoprecipitation studies show that VP35 is able to interact with the host proteins TRBP and PACT, which are part of the active RNA-induced silencing complex (RISC). TRBP and PACT are also regulators of PKR mediated translational shutdown. This suggests that VP35 can potentially target both RNAi and PKR related functions of TRBP and PACT. Sequence comparison of VP35 IID from different species of EBOV and MARV reveal a high degree of sequence similarity, especially in the functionally important regions, such as the first basic patch and central basic patch. Recent structural studies of Reston EBOV VP35 IID reveal a structure that is very similar to ZEBOV VP35 [30,31]. Collectively, these studies suggest that there could be a high degree of structural conservation of VP35 among the different filoviruses. How VP35 contributes to viral lethality is not well understood, but it is necessary for viral propagation because of its critical roles as host immune antagonist and as an essential cofactor for the viral RNA-dependent RNA polymerase. Therefore, VP35 is an attractive target for future panfiloviral therapeutic development due to its critical role in multiple functions.

### 3.2. EBOV VP24 Inhibits Transcription of Antiviral Genes

The matrix protein VP24 is unique to the *Filoviridae* family when compared with all other negative stranded RNA viruses such as *Paramyxoviridae*, *Rhabdoviridae* and *Bornaviridae* [32]. Previous experiments indicate that VP24 is a minor matrix protein present in viral particles [33,34]. VP24 promotes the formation of filamentous nucleocapsids that are comprised mainly of two other viral proteins, NP and VP35, and also appears to be required for formation of fully infectious virus particles [34–38]. VP24 plays a role in the transition from viral transcription and replication to virion assembly [39]. VP24 likely modulates host responses to infection and is also involved in host range expansion. VP24 acquires mutations during the experimental adaptation of EBOVs in mice and guinea pigs [22,40–42], which appear to be critical for enhanced EBOV virulence in these species. The main mechanism by which EBOV VP24 inhibits IFN signaling is through binding to karyopherin-α (KPN-α), which blocks nuclear accumulation of tyrosine-phosphorylated STAT1 [32,43,44].

**Figure 5.**
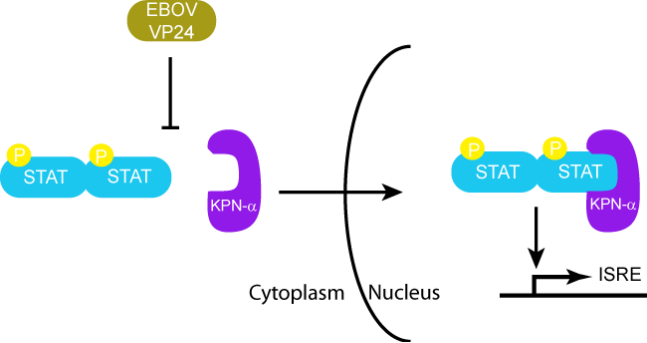
EBOV VP24 inhibits nuclear transport of phosphorylated STAT by KPN-α.

Initial studies that resulted in the identification of EBOV VP24 as a type I IFN antagonist were carried out in Vero cells, which can respond to but do not produce IFN-α/β. This screen utilized an IFN-responsive ISG54 promoter driving a reporter gene [44]. Among the EBOV proteins tested, only VP24 was able to significantly diminish ISG54 promoter activity. In addition, VP24 also inhibited IFN-γ mediated signaling suggesting that VP24 targets a cellular pathway that is common to both Type I and Type II IFNs. IFN-α/β induced signaling activates the JAK-STAT pathway, where STAT1 and STAT2 become phosphorylated, and the heterodimer translocates to the nucleus to activate antiviral ISGs. IFN-γ induced signaling results in homodimerization of phosphorylated STAT1 and nuclear translocation of the dimer, which activates gamma activated sequence (GAS) elements. However, when Vero cells were transfected with a plasmid encoding for VP24, it was observed that phospho-STAT1 localization to the nucleus was severely inhibited [44]. This was observed by using GFP-tagged STAT1 and visualizing its cellular localization in VP24 expressing cells. Similar results were obtained when Vero cells were infected with EBOV [32]. Monitoring of the phosphorylation of STAT1 in VP24 transfected cells using a phospho-STAT1 specific antibody showed that VP24 did not affect phosphorylation of STAT1. These results suggest that VP24 prevents the nuclear accumulation of phospho-STAT1, rendering EBOV infected cells resistant to IFNs (Figure 5). Further studies confirmed a direct interaction between VP24 and the KPN-α1 protein, which mediates shuttling of cargos such as STAT proteins and influenza virus nucleoprotein into the nucleus [45,46]. Immunoprecipitation studies in 293T cells transfected with VP24 showed that endogenous KPN-α1 coprecipitated with EBOV VP24 [32]. In addition, it was found that phosphorylated STAT1 and VP24 both bind to overlapping region of KPN-α1 (residues 425–538 that correspond to armadillo repeats 9 and 10 [32,47]) and that VP24 is able to disrupt the interaction of KPN-α1 with endogenous phospho-STAT1. Interestingly, both VP24 and STAT1 bind to KPN-α1 at an atypical site on the far C-terminus, whereas most proteins with a basic NLS enter the nucleus by binding KPN-α arm repeats 1– 8 [32,47]. Furthermore, VP24 also bound KPN-α5 and KPN-α6, two other members of the NPI-1 karyopherin subfamily, which are also bound by STAT1 [32]. Subsequent studies have also identified specific regions in VP24 which are critical for its interactions with KPN-α1. Using truncation mutants of VP24 to assess their ability to inhibit IFN mediated signaling, residues 26–50 and 142–146 were identified as functionally important. Specifically, residues W42 and K142 of VP24 were identified as critical residues, since mutation of one or both of these residues to alanine resulted in severe inhibition of its ability to interact with KPN-α1 [43]. All of the above data are consistent with a mechanism where VP24 inhibits STAT1 nuclear translocation by competing with STAT1 for the STAT1 binding site on KPN-α. However, other mechanisms, including VP24 altering KPN-α folding or trafficking, cannot be excluded. Although there is currently no high resolution structural information on EBOV VP24, bioinformatic analysis and *de novo* structure prediction using fragment based Rosetta method suggest that VP24 may be structurally similar to importin-α/β and exportin [48]. While this information suggests a potential mechanism involving mimicry of host transporter/cargo interactions, further studies will be necessary to delineate this and other mechanisms that may be mediated by EBOV VP24 to counter host immune responses.

### 3.3. MARV VP40 Inhibits the IFN-induced Phosphorylation of Jak and STAT Proteins

Despite the similarity in genome organization and sequence similarity between many of the EBOV and MARV encoded proteins, there are several important differences in terms of transcription, replication of genomic RNA, and overall pathogenesis [49]. While both EBOV and MARV share a similar genomic organization, their amino acid identity is low. For example MARV and EBOV VP24 and VP40 have approximately 35% amino acid similarity. This partially explains why MARV VP24 does not encode a similar antiviral function as Ebola VP24 [50]. Structural studies with EBOV VP40 show this viral matrix protein has two distinct domains. Both domains are structurally similar, consisting of a β-sandwich arranged as two sets of three antiparallel β-strands that stack on top of each other. There is a trypsin cleavage site in the loop region that connects the N-terminal and C-terminal domain. Upon proteolytic processing, the N-terminal domain of EBOV VP40 has been shown to form octamers in the presence of single-stranded RNA into ring-like structures [51–53]. These octameric structures have also been observed in cells infected with filoviruses, and hence thought to be important for the formation of new virions and viral budding from the host cell. However, there is no current structural information available for MARV VP40. Given the divergent sequences and potentially distinct functional roles by MARV and EBOV VP40 proteins, it is likely that the corresponding structure of MARV VP40 will reflect these differences.

**Figure 6.**
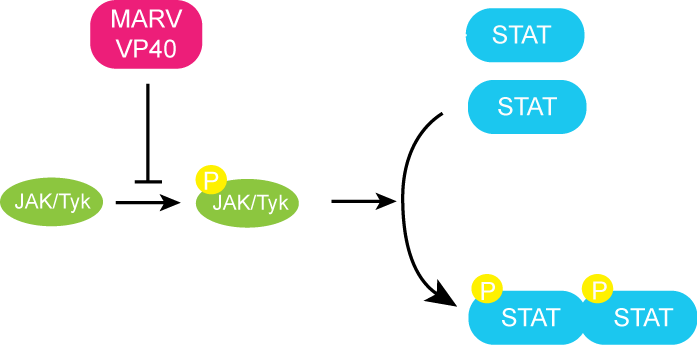
MARV VP40 inhibits JAK-STAT signaling. MARV VP40 inhibits the autophosphorylation of the JAK-1 kinase and subsequent phosphorylation of the STAT proteins.

Recent studies have shown that MARV VP40, in addition to its function as matrix protein in viral budding and egress, is sufficient to inhibit STAT phosphorylation (Figure 6) [50]. MARV infected cells, unlike those infected by EBOV, show severe inhibition of STAT1 and STAT2 phosphorylation [50]. Impairment of STAT phosphorylation was observed following treatment with type I IFN, type II IFN and IL-6 treatment, indicating that VP40 can block activation of multiple signaling pathways. The JAK-STAT pathway of normal cells is triggered upon treatment with IFN-α, resulting in STAT phosphorylation by the JAK family kinases. When MARV VP40 is expressed in these cells, STAT1 phosphorylation by JAK1 is inhibited [50]. Cumulatively, the available data suggest that Jak1 is a target of VP40, however the exact mechanism of this inhibition is unclear since no direct interactions between MARV VP40 and JAK1 has been demonstrated. This may suggest there are unidentified cellular proteins that facilitates the function of VP40. Moreover, no such evidence has been collected with EBOV VP40 to date, which may be explained by significant sequence differences between the two proteins. Future studies are needed to further characterize and compare structural and functional differences between MARV and EBOV VP40. Collectively these studies suggest that EBOV and MARV although phylogenetically similar, use distinct mechanisms to suppress the host immune response in addition to fundamental differences in transcriptional kinetics. Determining these differences and understanding their roles in pathogenesis and final outcome of disease is crucial in order to develop strategies for panfiloviral therapeutics.

## 4. Conclusions

Hemorrhagic fever outbreaks caused by filoviruses result in high fatality rates. The lack of approved therapeutics or vaccines along with the highly zoonotic nature of these viruses has made filoviruses a major health concern and a potential weapon of bioterrorism. In most filoviral infections, early innate immune responses are impaired, suggesting that virally encoded components are responsible for host immune evasion. Rapid progress in clinical research together with recent advances in biochemical and high resolution structural characterization of various filoviral proteins, as discussed above, are beginning to unravel some of the key host-viral interactions involved in this process. Ebola proteins VP35 and VP24 both impair innate immune responses. VP35 suppresses IFN production, while VP24 blocks nuclear translocation of phospho-STAT1, thereby preventing IFN mediated signaling. In contrast, MARV VP40, using a distinct mechanism, inhibits Jak and STAT phosphorylation, which also results in diminished host immunity during MARV infections. A more recent study suggests that other filoviral proteins, including EBOV VP30 and VP40, also counter the RNAi pathway [29]. While both EBOV and MARV retain similar genomic organization, variation in sequences has resulted in significant functional differences. Collectively, these studies highlight the complexity and diversity of immune evasion mechanisms used by filoviruses and point toward the need for detailed characterization of key host-viral interactions across different strains with varying host tropisms and pathogenesis. Future filoviral research aimed at combining the knowledge from regulatory mechanisms of host innate immunity with detailed characterization of viral components will likely provide critical knowledge that is needed for the development of prophylaxis and treatments against filoviruses.
